# On the Improvement of Wiener Attack on RSA with Small Private Exponent

**DOI:** 10.1155/2014/650537

**Published:** 2014-03-27

**Authors:** Mu-En Wu, Chien-Ming Chen, Yue-Hsun Lin, Hung-Min Sun

**Affiliations:** ^1^Department of Mathematics, Soochow University, Taipei, Taiwan; ^2^School of Computer Science and Technology, Shenzhen Graduate School, Harbin Institute of Technology, Shenzhen, China; ^3^Shenzhen Key Laboratory of Internet Information Collaboration, Shenzhen, China; ^4^CyLab, Carnegie Mellon University, Pittsburgh, PA 15213, USA; ^5^Department of Computer Science, National Tsing Hua University, Hsinchu, Taiwan

## Abstract

RSA system is based on the hardness of the integer factorization problem (IFP). Given an RSA modulus *N* = *pq*, it is difficult to determine the prime factors *p* and *q* efficiently. One of the most famous short exponent attacks on RSA is the Wiener attack. In 1997, Verheul and van Tilborg use an exhaustive search to extend the boundary of the Wiener attack. Their result shows that the cost of exhaustive search is 2*r* + 8 bits when extending the Weiner's boundary *r* bits. In this paper, we first reduce the cost of exhaustive search from 2*r* + 8 bits to 2*r* + 2 bits. Then, we propose a method named EPF. With EPF, the cost of exhaustive search is further reduced to 2*r* − 6 bits when we extend Weiner's boundary *r* bits. It means that our result is 2^14^ times faster than Verheul and van Tilborg's result. Besides, the security boundary is extended 7 bits.

## 1. Introduction

During the past 30 years, RSA [1] has been one of the most popular public-key cryptosystems worldwide. It has been widely used in several applications [2–4]. The security of RSA is often based on the hardness of the integer factorization problem (IFP), which remains a well-studied problem [5, 6]. Current RSA standards suggest that an RSA modulus *N*  should be at least 1024 bits long. Using the best-known factoring algorithms, the expected workload of factoring a 1024 bit modulus is 2^80^, which is currently believed to be infeasible. However, although the use of a large RSA modulus achieves a high security level, the encryption and decryption procedures involve heavy exponential modular multiplications, which make RSA inefficient. Therefore, many approaches have been investigated for speeding-up the RSA encryption (or signature-verification) and RSA decryption (or signature-signing) [7–12]. Furthermore, since the signing task is often executed by lightweight devices, such as smart cards, mobile phones, or PDAs, the research on speeding-up signature-signing is more practical and important.

The most popular method for reducing the signing time is to apply a small private exponent *d* since the complexity of signing depends on the bit-length of *d*. In order to achieve this goal, the order of choosing *e* and *d* is exchanged. *d* is first chosen in the RSA-key generation algorithm, and the corresponding public exponent *e* satisfying *ed* ≡ 1(mod⁡*φ*(*N*)) is then calculated. These RSA variants are called RSA-Small-*d*. Nevertheless, the variants of RSA-Small-*d* have the security flaws [13–18]. In fact, instances of RSA with *d* < *N* ^1/4^ can be efficiently broken by Wiener attack [16]. Besides, Boneh and Durfee's lattice-based attack [19] indicated that an instance of RSA-Small-*d* with *d* < *N* ^0.292^ should be considered to be an unsafe system.

In 1997, Verheul and van Tilborg [20] used an exhaustive search to further extend the boundary of the Wiener attack. Suppose *r* = log⁡_2_⁡*d* − log⁡_2_⁡*N* ^1/4^; their result shows that an exhaustive search for 2*r* + 8 bits is required to extend the Wiener's boundary *r* bits. Assume that an exhaustive search for 64 bits is feasible in terms of current computational abilities; solving *r* for the equation “2*r* + 8 = 64” yields *r* = 28, which implies that the boundary of the Wiener attack should be raised up to *N* ^1/4^2^28^.

In this paper, we attempt to reduce the cost of exhaustive search of Verheul and van Tilborg's result. We propose an approach to reduce the cost of exhaustive search when we desire to extend Wiener's boundary. This approach includes two steps.

**Step 1**

We investigate a method for searching as many MSBs (most significant bits) of *p* + *q* as possible, which is equivalent to estimating *p* + *q* as accurately as possible. In this step, to extend Wiener's boundary *r* bits, an exhaustive search requires 2*r* + 2 bits. It means that our result is better than Verheul and van Tilborg's cost, which requires an exhaustive search for 2*r* + 8 bits.

**Step 2**

We develop an approach, called “Estimated Prime Factor (EPF),” to estimate *p* + *q*, and then we derive two integers *p*_*E*_ and *q*_*E*_, which are the estimations of *p* and *q*, respectively. Using EPF, the first 8 MSBs of *p* + *q* can be determined. This result is more accurate than the traditional estimation, which estimates *p* + *q* by $2\sqrt{N}$. Applying EPF can further reduce the cost of exhaustive search. More specifically, to extend Wiener's boundary *r* bits, an exhaustive search requires 2*r* − 6 bits. As compared to Verheul and van Tilborg's result, which requires an exhaustive search for 2*r* + 8 bits, the security boundary is extended 7 bits.

### 1.1. Our Contribution

The contributions of this paper are summarized as follows.

1. We first reduce the cost of exhaustive search from 2*r* + 8 (Verheul and van Tilborg's result) bits to 2*r* + 2 bits when we extend Wiener's boundary *r* bits. It means that exhaustive search is 2^6^ times faster in Step 1. Besides, the security boundary is extended 3 bits.
2. We propose a novel approach, named EPF, for estimating the prime factors of *N*. With EPF, the cost of the exhaustive search for 2*r* + 2 bits (mentioned in contribution (1)) is further reduced to 2*r* − 6 bits. Compared with Verheul and van Tilborg's result, exhaustive search is 2^14^ times faster. Besides, the security boundary is extended 7 bits.

### 1.2. Organization

The remainder of this paper is organized as follows.  Section 2 presents the preliminaries of this paper.  Section 3 describes Step 1 of our approach. In Section 4, we propose the EPF to estimate the prime factors of an RSA modulus. Next, Step 2 of our approach which is applying EPF is proposed in Section 5. Finally, we present our conclusions and future works in Section 6.

## 2. Preliminary

In this section, we introduce the preliminaries of this paper which include RSA and its variants and the Wiener attack.

### 2.1. RSA and Its Variants

The RSA cryptosystem [1] consists of three parts, RSA-key generation, encryption, and decryption which are described as follows.

#### 2.1.1. RSA-Key Generation, Encryption, and Decryption

The RSA-key generation outputs the RSA key: (*N*, *e*, *d*). First, randomly choose two large prime numbers *p* and *q* and compute *N* = *pq*, where *N* is called RSA modulus. Secondly, let *e*, called public exponent, be a randomly chosen integer such that gcd (*e*, *φ*(*N*)) = 1, where *φ*(·) is Euler's phi function. Then, let *d*, called private exponent, be the multiplicative inverse modulo *φ*(*N*) (i.e., *ed* ≡ 1  (mod⁡ *φ*(*N*))). The pair (*e*, *N*) is the public key and the pair (*d*, *N*) is the private key.

From the key relation *ed* ≡ 1  (mod⁡ *φ*(*N*)), there exists a unique positive integer *k* satisfying

(1) $$\begin{array}{c} ed=1+k\cdot \varphi \left(N\right). \end{array}$$

We call (1) as the RSA-key equation. To encrypt a plaintext message *M* ∈ *ℤ*_*N*_, compute *C* ≡ *M* ^*e*^(mod⁡*N*). The result *C* is called the ciphertext of *M*. To execute RSA decryption, a ciphertext *C* ∈ *ℤ*_*N*_ is decrypted by raising it to the *d*th power modulo *N*. From Lagrange's theorem, it follows that

(2) $$\begin{array}{c} C^{d}\left(\underset{}{\mathrm{mod}}N\right)=M^{ed}\left(\underset{}{\mathrm{mod}}N\right)\equiv M\left(\mathrm{mod}N\right)=M. \end{array}$$

Usually, one often selects *e* as small as possible due to the reason of efficient encryption (or signature-verification). The smallest *e* is suggested to be 2^32^ + 1 rather than 2^16^ + 1 while a known affine relation between two messages exists [21]. We call the RSA system with small public exponent *e* as “RSA-Small-*e*.” On the other hand, since the cost of decryption (or signature-signing) can be significantly reduced when the private exponent *d* is much smaller than *φ*(*N*), in order to simply reduce the decryption (or signature-signing) time, one can select a small private exponent *d* first in RSA-key generation. Such variant is called RSA-Small-*d*, which is shown in the following.

#### 2.1.2. RSA-Small-*d*

Generating instances of RSA with a small private exponent is easy with the observation that the RSA-key equation (1) is symmetric with respect to the public and private exponents. We simply follow the same key generation of original RSA but exchange the choosing order of public and private exponents.

One of the drawbacks of RSA-Small-*d* is its inefficient encryption. Since the public exponent *e* in RSA-Small-*d* is always computed as the inverse of *d* modulo *φ*(*N*), it is expected with high probability that *e* will be almost the same size as *φ*(*N*). In conclusion, RSA-Small-*d* saves the decryption (or signature) cost while the encryption cost remains large.

### 2.2. The Wiener Attack

One of the most famous short exponent attacks on RSA is the Wiener attack. Boneh and Durfee [22] showed in 1990 that RSA-Small-*d* should be considered insecure when *d* < *N* ^1/4^. He achieved the attack through the technique of continued fractions. In the following paragraph, we briefly introduce the continued fractions and the Weiner attack. The details can be referenced in [16].

**Definition 1 (continued fractions)**

For any positive real number *α*, define *α* = *ξ*_0_, *a*_*i*_ = ⌊*ξ*_*i*_⌋, *ξ*_*i*+1_ = 1/(*ξ*_*i*_ − *a*_*i*_) for *i* = 0, 1, 2, …. Then *α* can be expanded into the following form:

(3) $$\begin{array}{c} \alpha_{i}=a_{0}+1/\left(a_{1}+1/\left(a_{2}+1/\left(a_{3}+1/\cdots \right)\right)\right). \end{array}$$

The form of (3) is called the continued fraction expression of *α*. For simplicity, we write (3) to be *α* = (*a*_0_, *a*_1_, *a*_2_,…). In addition, denote *α*_*i*_ = (*a*_0_, *a*_1_,…, *a*_*i*_) as the *i*th convergent of the continued fraction expansion of *α*, which means

(4) $$\begin{array}{c} \alpha_{i}=a_{0}+1/\left(a_{1}+1/\left(a_{2}+1/\left(\cdots +1/a_{i}\right)\right)\right). \end{array}$$

If *α* is a rational number, then the process of computing its continued fraction expression, see (3), will cease in some index *k*. That is, *α* = *α*_*k*_. If *α* is irrational, then the process will go on unceasingly.

**Theorem 2**

Denote *h*_*i*_/*k*_*i*_ as the fraction form of (4); that is, *h*_*i*_/*k*_*i*_ = *α*_*i*_, where *h*_*i*_ and *k*_*i*_ are positive integers. Then, *h*_*i*_ and *k*_*i*_ can be calculated by defining *h*_−2_ = 0, *k*_−2_ = 1, *h*_−1_ = 1, and *k*_−1_ = 0. And *h*_*i*_ = *a*_*i*_ *h*_*i*−1_ + *h*_*i*−2_ and *k*_*i*_ = *a*_*i*_ *k*_*i*−1_ + *k*_*i*−2_, for *i* ≥ 0.

Following the notations in Theorem 2, we have Corollary 3.

**Corollary**

For any *i* ≥ 1,

(5) $$\begin{array}{c} \left|\alpha -\frac{h_{i+1}}{k_{i+1}}\right|<\left|\alpha -\frac{h_{i}}{k_{i}}\right|. \end{array}$$

Furthermore, if *α* is an irrational number, then lim⁡_*i*→*∞*_ *h*_*i*_/*k*_*i*_ = *α*.

**Theorem 4**

If a real number *α* and a reduced fraction *a*/*b* satisfy

(6) $$\begin{array}{c} \left|\alpha -\frac{a}{b}\right|<\frac{1}{2b^{2}}, \end{array}$$

then *a*/*b* equals to one of the convergents of the continued fraction expression of *α*.

#### 2.2.1. The Wiener Attack. 

The Wiener attack [16] is based on approximations using continued fractions to find the private exponent of RSA-Small-*d* in polynomial time if *d* < *N* ^1/4^, where *p* and *q* are of the same bit-length. Note that the RSA-key equation, *ed* = 1 + *k* · *φ*(*N*), can be rewritten as

(7) $$\begin{array}{c} \left|\frac{e}{\varphi \left(N\right)}-\frac{k}{d}\right|=\left|\frac{1}{d\varphi \left(N\right)}\right|, \end{array}$$

which is similar to the form of the left-hand side of (6). In order to apply Theorem 4, we replace *e*/*φ*(*N*) of (7) by *e*/*N*, which is known for everyone, and set the difference between *e*/*N* and *k*/*d* to be smaller than 1/2*d* ^2^; that is,

(8) $$\begin{array}{c} \left|\frac{e}{N}-\frac{k}{d}\right|<\frac{1}{2d^{2}}. \end{array}$$

Therefore, according to Theorem 4, *k*/*d* can be found by computing one of the convergents of the continued fraction expression of *e*/*N*.

The security boundary of the Wiener attack is deduced from the sufficient condition for (8). Since $p\approx q\approx \sqrt{N}$ and *k* ≈ *d*, the left-hand side of (8) is simplified to

(9) $$\begin{array}{c} \left|\frac{e}{N}-\frac{k}{d}\right|=\frac{k\left(p+q-1\right)-1}{Nd}\approx \frac{2\sqrt{N}}{N}=\frac{2}{\sqrt{N}}. \end{array}$$

Hence, (8) is transformed to

(10) $$\begin{array}{c} \frac{2}{\sqrt{N}}<\frac{1}{2d^{2}}, \end{array}$$

which gives the security boundary of the Wiener attack (after ignoring the constant term):

(11) $$\begin{array}{c} d<N^{1/4}. \end{array}$$

### 2.3. Verheul and van Tilborg's Extension

The Wiener attack works very well and efficiently when the private exponent *d* < *N* ^1/4^. However, what about if the bit-length of *d* is slightly larger than the bit-length of *N* ^1/4^? In 1997, Verheul and van Tilborg [20] proposed a technique to solve this problem by performing an exhaustive search for 2*r* + 8 bits, where *r* = log⁡_2_ *d* − log⁡_2_ *N* ^1/4^ means that the bit-length of *d* is longer than the bit-length of *N* ^1/4^ by *r* bits.

Verheul and van Tilborg observed that *k*/*d* in (8) can be represented as follows:

(12) $$\begin{array}{c} \frac{k}{d}=\frac{{}_{p_{j+1}}U+{\left(U\Delta +V\right)}_{p_{j}}}{{}_{q_{j+1}}U+{\left(U\Delta +V\right)}_{q_{j}}}, \end{array}$$

where *p*_*i*_/*q*_*i*_ is the *i*th convergent of the continued fraction expression of *e*/*N*, Δ = 1 or 2, and *U* and *V* are two unknown integers with upper bounds as follows:

(13) $$\begin{array}{c} \log_{2}U\leq r+4,\;\;\log_{2}V\leq r+4. \end{array}$$

Since Δ is a small integer, we can omit its uncertainty. The unknown parts of (12) are about 2*r* + 8 bits, which give the result of Verheul and van Tilborg's extension: extending Wiener's boundary by *r* bits requires an exhaustive search for about 2*r* + 8 bits.

Assume that an exhaustive search for 64 bits is feasible in terms of the current computational capabilities. Solving *r* for the equation “2*r* + 8 = 64” yields *r* = 28, which implies that Wiener's boundary can be extended 28 bits over the bit-length of *N* ^1/4^. Therefore, RSA-Small-*d* with *d* < *N* ^1/4^2^28^ can be totally broken by continued fraction attack plus the cost of performing an exhaustive search for 64 bits. In Section 3, we show that, in order to extend Wiener's boundary by *r* bits, it requires only an exhaustive search for 2*r* + 2 bits, rather than that from Verheul and van Tilborg's extension for cost, which requires an exhaustive search for 2*r* + 8 bits.

## 3. Reducing the Cost of Exhaustive Search to 2*r* + 2 Bits

Our approach contains two steps which are described in Sections 3 and 5, respectively. In this section, we investigate a method for searching as many MSBs (most significant bits) of *p* + *q* as possible, which is equivalent to estimating *p* + *q* as accurately as possible. With this method, we can reduce the cost of exhaustive search from 2*r* + 8 bits (Verheul and van Tilborg's extension) to 2*r* + 2 bits when we extend Wiener's boundary *r* bits.

Let *A* be the estimation of *p* + *q*. Throughout this paper, we assume *A* < *p* + *q*. Thus *φ*(*N*) = (*N* + 1)−(*p* + *q*) is estimated as (*N* + 1) − *A*, which implies

(14) $$\begin{array}{c} \frac{e}{\varphi \left(N\right)}\approx \frac{e}{\left(N+1\right)-A}. \end{array}$$

Applying (14) to the Wiener attack, that is, replacing *e*/*N* of (8) by *e*/((*N* + 1) − *A*), we have

(15) $$\begin{array}{c} |\frac{e}{N+1-A}-\frac{k}{d}|<\frac{1}{2d^{2}}. \end{array}$$

Note that if *A* = *p* + *q*, then (15) always holds for any *d* because

(16) |eN+1−(p+q)−kd|=|ed−k(N+1−(p+q))(N+1−(p+q))d|=1φ(N)d<12d2.

Simplifying (15) yields

(17) |eN+1−A−kd|=|ed−k(N+1−A)(N+1−A)d|=k[(p+q)−A]−1(N+1−A)d<12d2,

which is

(18) $$\begin{array}{c} 2dk\left[\left(p+q\right)-A\right]-2d<N+1-A. \end{array}$$

Solving *d* in (18), we get the upper bound of the private exponent:

(19) $$\begin{array}{c} d<\frac{N+1-A}{2k\left(p+q-A\right)-2}. \end{array}$$

According to the above inequality, we know that the smaller the difference between *p* + *q* and *A*, the higher the upper bound of *d*. Consequently, in order to extend the security boundary of RSA-Small-*d*, we attempt to estimate *A* as precisely as possible such that *p* + *q* − *A* becomes small. Equation (19) also shows that the complexity of further extending Wiener's boundary can be reduced to the complexity of estimating the MSBs of *p* + *q*. The relation is shown in the following.

Rearranging (18) we have

(20) $$\begin{array}{c} 2dk\left(p+q-1\right)-2d<N+\left(2dk-1\right)\left(A-1\right). \end{array}$$

Denote Λ as the difference between *p* + *q* and *A*. That is, Λ = *p* + *q* − *A*. Replacing *A* in (20) by *p* + *q* − Λ  conducts

(21) 2dk(p+q−1)−2d<N+(2dk−1)((p+q−Λ)−1)=2dk(p+q−1) +φ(N)−Λ(2dk−1).

In (21), eliminating 2*dk*(*p* + *q* − 1) in both sides we get

(22) $$\begin{array}{c} \Lambda \left(2dk-1\right)-2d<\varphi \left(N\right). \end{array}$$

Now we consider the bit-length of each side. Assume that the bit-length of *d* is *n*/4 + *r* bits, which is longer than Wiener's boundary by *r* bits. Due to the key generation of RSA-Small-*d*, the parameter *k* is almost the same size as *d* with a high probability; that is, log⁡_2_ *k* ≈ log⁡_2_ *d*. In addition, we perform an exhaustive search for the first *s* MSBs of *p* + *q*. Thus the difference between *p* + *q* and *A* can be reduced to (*n*/2 + 1) − *s* bits; that is, log⁡_2_Λ ≈ (*n*/2 + 1) − *s*. Consequently, The term Λ · 2*dk*, which dominates the size in the left-hand side of (22), is about ((*n*/2 + 1) − *s*) + 1 + 2 × (*n*/4 + *r*) bits long and the sufficient condition of (22) is

(23) $$\begin{array}{c} \underset{\text{for}\;\Lambda }{\underset{︸}{\left((n/2+1)-s\right)}}+\underset{\text{for}\;2dk}{\underset{︸}{1+2\times \left(n/4+r\right)}}<n, \end{array}$$

which is simplified to

(24) $$\begin{array}{c} 2r+2<s. \end{array}$$

Equation (24) gives the following conclusion. In order to extend Wiener's boundary by *r* bits, we have to perform an exhaustive search for the first 2*r* + 2 MSBs of  *p* + *q*, where *r* = log⁡_2_ *d* − log⁡_2_ *N* ^1/4^. This result is better than that of Verheul and van Tilborg's cost [20], which requires an exhaustive search for 2*r* + 8 bits. Therefore, assume that an exhaustive search for 64 bits is feasible in terms of current computational abilities. Solving *r* for

(25) $$\begin{array}{c} 2r+2=64 \end{array}$$

yields *r* = 31, which means that RSA-Small-*d* is insecure when *d* < *N* ^1/4^2^31^.

## 4. Estimated Prime Factor (EPF)

In this section, a novel approach called Estimated Prime Factor (EPF), which is used to estimate the prime factors of an RSA modulus *N*, is proposed.

### 4.1. EPF

Without loss of generality, we assume that *q* < *p* < 2*q*, where *N* = *pq*. Denote *D*_*p*_ and *D*_*q*_ as the distances between $\sqrt{N}$ & *p* and *q* & $\sqrt{N}$, respectively. That is,

(26) $$\begin{array}{c} p=\sqrt{N}+D_{p},\;\;q=\sqrt{N}-D_{q}. \end{array}$$

Applying (26) to *N* = *pq* yields

(27) N=p·q=(N+Dp)·(N−Dq)

(28) =N+N·(Dp−Dq)−Dp·Dq.

Eliminating *N* in both sides of (27) we have

(29) $$\begin{array}{c} D_{p}\cdot D_{q}=\sqrt{N}\cdot \left(D_{p}-D_{q}\right), \end{array}$$

which leads to

(30) $$\begin{array}{c} \frac{1}{\sqrt{N}}=\frac{D_{p}-D_{q}}{D_{p}D_{q}}. \end{array}$$

Equation (30) is quite interesting because the irrational fraction $1/\sqrt{N}$ reveals partial information of *D*_*p*_ − *D*_*q*_ and *D*_*p*_ · *D*_*q*_. Note that with *D*_*p*_ − *D*_*q*_ and *D*_*p*_ · *D*_*q*_ we can compute *D*_*p*_ + *D*_*q*_ by

(31) $$\begin{array}{c} {\left(D_{p}+D_{q}\right)}^{2}={\left(D_{p}-D_{q}\right)}^{2}+4D_{p}D_{q} \end{array}$$

and solve *D*_*p*_ and *D*_*q*_ as follows:

(32) $$\begin{array}{c} D_{p}=\frac{D_{p}+D_{q}}{2}+\frac{D_{p}-D_{q}}{2},\\ D_{q}=\frac{D_{p}+D_{q}}{2}-\frac{D_{p}-D_{q}}{2}. \end{array}$$

Now we use continued fractions to construct a rational sequence to approximate $1/\sqrt{N}$. Suppose that the *i*th convergent of the continued fraction expansion of $1/\sqrt{N}$ is *h*_*i*_/*k*_*i*_. According to Theorem 2, we know that

(33) $$\begin{array}{c} \frac{h_{i}}{k_{i}}⟶\frac{1}{\sqrt{N}},\;\text{as}\;\;i⟶\infty . \end{array}$$

Since the sizes of *h*_*i*_ and *k*_*i*_ grow with increase of the index *i* (see Theorem 2), there exists an index *t* such that

(34) $$\begin{array}{c} h_{t}<D_{p}-D_{q}<h_{t+1}. \end{array}$$

We use *h*_*t*_ and *k*_*t*_ as the estimations of *D*_*p*_ − *D*_*q*_ and *D*_*p*_ *D*_*q*_, respectively, instead of using the larger ones. That is,

(35) $$\begin{array}{c} h_{t}\approx D_{p}-D_{q},\;\;k_{t}\approx D_{p}D_{q}. \end{array}$$

From (31), *D*_*p*_ + *D*_*q*_ is estimated as

(36) $$\begin{array}{c} D_{p}+D_{q}\approx \sqrt{h_{t}^{2}+4k_{t}}. \end{array}$$

And thus *D*_*p*_ and *D*_*q*_ are estimated as

(37) $$\begin{array}{c} D_{p}\approx \frac{\sqrt{h_{t}^{2}+4k_{t}}+h_{t}}{2},\;\;D_{q}\approx \frac{\sqrt{h_{t}^{2}+4k_{t}}-h_{t}}{2}. \end{array}$$

Finally, we define the estimated prime factors of *N* as

(38) $$\begin{array}{c} p_{E}:=\left\lceil \sqrt{N}+\frac{\sqrt{h_{t}^{2}+4k_{t}}+h_{t}}{2}\right\rceil ,\\ q_{E}:=\left\lfloor \sqrt{N}-\frac{\sqrt{h_{t}^{2}+4k_{t}}-h_{t}}{2}\right\rfloor . \end{array}$$

### 4.2. Theoretical Estimation and Experimental Result on Searching the Index *t*

The process of computing the convergent of the continued fraction expression of $1/\sqrt{N}$ should be ceased at the index *t* satisfying (34). Thus, we have to estimate the size of *D*_*p*_ − *D*_*q*_ in order to determine the index *t*. Since *D*_*p*_ < *p* and *D*_*q*_ < *q*, *h*_*t*_ should not be set larger than *n*/2 bits at least. Next, we investigate the method to estimate the index *t* theoretically and experimentally.

#### 4.2.1. Theoretical Estimation

From the definitions of *D*_*p*_ and *D*_*q*_ in (26), we have

(39) $$\begin{array}{c} D_{p}-D_{q}=p+q-2\sqrt{N}={\left(\sqrt{p}-\sqrt{q}\right)}^{2}, \end{array}$$

which is equivalent to

(40) $$\begin{array}{c} \log_{2}\left(D_{p}-D_{q}\right)=2\log_{2}\left(\sqrt{p}-\sqrt{q}\right). \end{array}$$

Equation (40) shows that the bit-length of *D*_*p*_ − *D*_*q*_ is twice the bit-length of $\sqrt{p}-\sqrt{q}$. Consider the following problem.

*Problem*. Randomly select two prime numbers *p* and *q* of *n*/2 bits; what is the expected value of the number of MSBs of $\sqrt{p}$ and $\sqrt{q}$ that are identical?

From our theoretical estimation, the expected value is about 2.6, and it is almost independent of the bit-length of *N*. This implies that, for any two randomly selected prime numbers *p* and *q* of *n*/2 bits each, the first 2.6 MSBs of $\sqrt{p}$ and $\sqrt{q}$ are identical on average. Consequently, according to (40), the size of *D*_*p*_ − *D*_*q*_ is expected to be 2 × (*n*/4 − 2.6) = *n*/2 − 5.2 bits, which increases linearly with the bit-length of *N*.

#### 4.2.2. Experimental Results

Table 1 shows the experimental results for the index *t* in EPF. Suppose that *p* and *q* are two randomly generated prime numbers of *n*/2 bits each; we then compute log⁡_2_(*D*_*p*_ − *D*_*q*_), log⁡_2_(*h*_*t*_), and log⁡_2_(*h*_*t*+1_), which denote the bit-lengths of *D*_*p*_ − *D*_*q*_, *h*_*t*_, and *h*_*t*+1_, respectively. Each block in the table is evaluated from the average value of 1000 experimental instances. As can be observed from the first row, the bit-length of *D*_*p*_ − *D*_*q*_ is approximately equal to (*n*/2 − 7) bits long for all *n* and is greater than that of *h*_*t*_ by at least 1  bit on average. This result is slightly different from the result in the previous version at* ACNS*'*07* [23] due to the reason of using different samples in the experiments. Note that in this paper we implement EPF with uniformly distributed samples which are more objective. Moreover, the values of log⁡_2_(*D*_*p*_ − *D*_*q*_) in Table 1 are slightly smaller than the theoretical estimation *n*/2 − 5.2 bits; the reason may be that we ignore the usage of prime-counting function *π*(·) in the calculation. However, the values in Table 1 actually increase linearly with the bit-length of *N*.

In EPF, we simply estimate the value of *D*_*p*_ − *D*_*q*_, which is, however, smaller than the actual value. On the other hand, up to now, there is no theory to justify the difference between the bit-lengths of *h*_*t*_ and *D*_*p*_ − *D*_*q*_; in fact, this would be an interesting subject of inquiry.

### 4.3. Accuracy and Further Improvement

We demonstrate the accuracy of EPF in Table 2. Each entry in the table is the data averaged over 1000 samples. The first row shows the difference of the bit-length between *p* + *q* and its estimation by using $2\sqrt{N}$. The second row shows the difference of the bit-length between *p* + *q* and its estimation by using EPF. As can be seen in Table 2, using *p*_*E*_ + *q*_*E*_ as the estimation is more accurate than using $2\sqrt{N}$ at least one bit on average. This result shows that EPF is better than the traditional estimation method.

To further raise the accuracy rate of EPF, we may employ the properties of continued fractions. According to Theorem 2, we know that

(41) $$\begin{array}{c} h_{t+1}=a_{t}h_{t}+h_{t-1},\;\;k_{t+1}=a_{t}k_{t}+k_{t-1}, \end{array}$$

where *a*_*t*_ is the *t*th component of the continued fraction expression of $1/\sqrt{N}$ (see Definition in Section 2.2). Consequently, for any real number *λ* ∈ [1, *a*_*t*_], we have

(42) $$\begin{array}{c} h_{t}<\lambda h_{t}+h_{t-1}<h_{t+1},\;\;k_{t}<\lambda k_{t}+k_{t-1}<k_{t+1}. \end{array}$$

Since *D*_*p*_ − *D*_*q*_ and *D*_*p*_ · *D*_*q*_ are also in the intervals (*h*_*t*_, *h*_*t*+1_) and (*k*_*t*_, *k*_*t*+1_), respectively, *λh*_*t*_ + *h*_*t*−1_ and *λk*_*t*_ + *k*_*t*−1_ might be better estimations of *D*_*p*_ − *D*_*q*_ and *D*_*p*_ · *D*_*q*_. Hence, an interesting question would be how to find a suitable value of *λ* that yields better estimations of *D*_*p*_ − *D*_*q*_ and *D*_*p*_ · *D*_*q*_. Note that, from the properties of continued fractions, we have

(43) $$\begin{array}{c} \frac{h_{t+1}}{k_{t+1}}>\frac{1}{\sqrt{N}}>\frac{h_{t}}{k_{t}}\;\text{if}\;t\;\text{is old,}\\ \frac{h_{t+1}}{k_{t+1}}<\frac{1}{\sqrt{N}}<\frac{h_{t}}{k_{t}}\;\text{if}\;t\;\text{is even}. \end{array}$$

Equation (43) implies that there exists an irrational number *λ*_1_, such that

(44) $$\begin{array}{c} \frac{\lambda_{1}h_{t}+h_{t-1}}{\lambda_{1}k_{t}+k_{t-1}}=\frac{1}{\sqrt{N}}. \end{array}$$

To find an appropriate number *λ*, one method could be to choose *λ*, which is very close to *λ*_1_, which might yield better estimations of *D*_*p*_ − *D*_*q*_ and *D*_*p*_ · *D*_*q*_. However, we leave this concept as the subject of future work on EPF.

## 5. Applying EPF to Reduce the Cost of Exhaustive Search to 2*r* − 6 Bits

In this section, we apply EPF proposed in Section 4 to further reduce the cost of exhaustive search.

From the results of Section 3, the security boundary of RSA-Small-*d* depends on the known MSBs of *p* + *q*. In EPF, the experimental results show that the 1st to the 8th MSB of *p* + *q*, denoted as MSB_1^∼^8_(*p* + *q*), can be correctly determined with high probability (see Table 2). Consequently, setting *p*_*E*_ + *q*_*E*_ = 2^(*n*/2+1)−8^ *A*_1_ + *A*_2_, where *A*_2_ < 2^*n*/2−7^, then

(45) $$\begin{array}{c} {\left(A_{1}\right)}_{2}=\text{MS}{\text{B}}_{1^{∼}8}\left(p+q\right), \end{array}$$

where (*A*_1_)_2_ denotes the binary representation of *A*_1_. Setting Λ = (*p* + *q*) − (*p*_*E*_ + *q*_*E*_), (45) also shows that Λ is about (*n*/2 + 1) − 8 bits long. Hence, representing (22) according to the bit-length of the items, Λ, *d*, *k*, and *φ*(*N*) yields

(46) $$\begin{array}{c} \left(\left(\frac{n}{2}+1\right)-8\right)+1+2\left(\frac{n}{4}+r\right)<n. \end{array}$$

Moreover, by performing an exhaustive search for *s* bits after the 8th MSB of *p* + *q*, that is, MSB_9^∼^8+*s*_(*p* + *q*), we can further reduce the size of Λ to (*n*/2 + 1) − (8 + *s*) bits. This implies that the 1st to the (8 + *s*)th MSB of *p* + *q* can be correctly determined and the size of Λ is reduced to (*n*/2 + 1) − (8 + *s*) bits. Hence, (46) is revised to

(47) $$\begin{array}{c} \left(\frac{n}{2}+1\right)-\left(8+s\right)+1+2\left(\frac{n}{4}+r\right)<n, \end{array}$$

which is simplified to

(48) $$\begin{array}{c} 2r-6<s. \end{array}$$

Equation (48) is the improved result when applying EPF to the method presented in Section 3. As a conclusion, extending Wiener's boundary by *r* bits requires only an exhaustive search for 2*r* − 6 bits, which results in a lower computational cost than that with Verheul and van Tilborg's extension. We summarize the improvements in each type of attack in Table 3.

**Figure 1:**
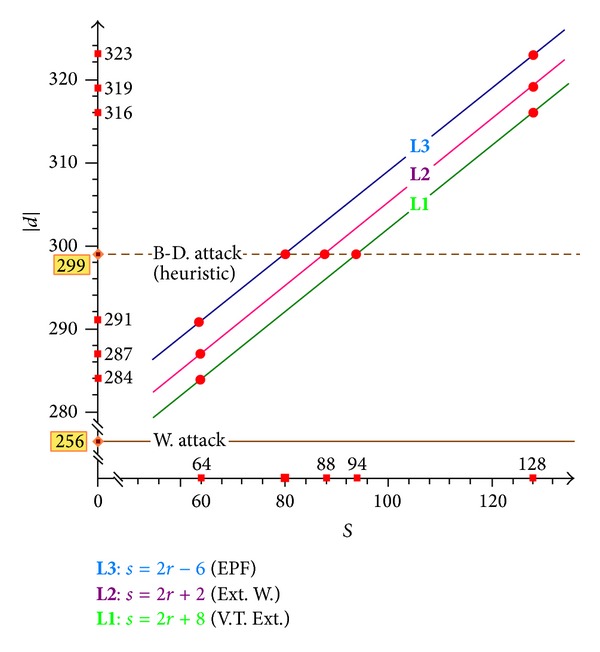
The boundaries of the extensions of the Wiener attack under different computational capabilities, where 256 and 299 are the boundaries of the Wiener attack (W. Attack) and Boneh and Durfee's attack (B-D. Attack), respectively. **L**1, **L**2, and **L**3 denote the boundaries of Verheul and Tilborg's extension (V-T. Ext.) (see [20]), the extension of the Wiener attack (Step 1) (Ext. W.) (see (24)), and the extension of the Wiener attack through EPF (EPF) (see (48)).

With the progress of technology, the ability of machines to perform exhaustive searches will only increase.  Figure 1 shows the relations between the security boundaries of the extensions of the Wiener attack and machines with different computational abilities. The symbol *s* denotes the required number of bits for an exhaustive search to extend Wiener's boundary, and the symbol |*d*| denotes the upper bound of the insecure private exponent. In terms of the current computational capabilities, an exhaustive search for 64 bits is feasible. Hence, the lines **L**1, **L**2  and  **L**3 yield the improvements of 28 bits, 31 bits, and 35 bits, respectively, over Wiener's boundary. The boundaries of the extensions of the Wiener attack (see V-T. Ext., Ext. W., and EPF in Figure 1) can be raised to 284 bits, 287 bits, and 291 bits, respectively, when the RSA modulus *N* is 1024 bits long. Furthermore, if an exhaustive search for 80 bits is feasible, the upper bound of the extension of the Wiener attack through EPF is raised to *N* ^1/4^2^43^, which is 299 bits when *N* is 1024 bits long (see **L**3: EPF). This result is comparable to the boundary of the lattice attack proposed by Boneh and Durfee [19], which has a best upper bound, but heuristic, at the present. Note that there is no guaranty that a heuristic algorithm can output the solution. One may concern whether the assumption that an exhaustive search for 80 bits is feasible or not. In the opinion of current development, it will not be a difficult task to achieve such computational capability in the near future. According to Moore's Law, computers will double in speed approximately every 18 months, which further supports our assumption. Moreover, paralleling techniques and special-purpose machines can help in speeding-up the computation.

## 6. Conclusion and Future Works

With the rapid growth of different network environments such as wireless sensor networks [24–27], security is normally the most concerned issue. In this paper, we propose a method, called EPF, to estimate the prime factors of an RSA modulus. With EPF, the cost of exhaustive search can further reduce to 2*r* − 6 bits. It means that the cost is 2^14^ times faster than Verheul and van Tilborg's result and the security boundary is extended 7 bits. It should be noted that their method for an exhaustive search is heuristic since this method is based on the results of distribution of small partial quotient in the continued fraction expansions.

An interesting problem in EPF is whether there exists a deterministic algorithm for finding an index *t* satisfying *h*_*t*_ < *D*_*p*_ − *D*_*q*_ < *h*_*t*+1_. In this paper, we use the theoretical estimation to determine the index *t*. The success rate is 85.1% according to our experiments. Now, another question arises—how to increase the success rate of the process of finding the index *t* when the deterministic algorithm is not developed. In addition, the other researchable question is how to improve the accuracy rate of MSBs of *p*_*E*_ + *q*_*E*_, which brings a greater contributive effort of EPF.

We should point out that EPF can be applied to Dujella's refinement [14] and the generalized Wiener attack [18]. Moreover, we foresee that EPF could be applied to other cryptogrammic aspects, especially to the attacks for cryptosystems based on the integer factorization problem (IFP). For example, the lattice technique is commonly used for the cryptanalysis of RSA [17, 28–30] or for the attacks on RSA with small exponents [15, 18, 19, 21, 22, 31, 32]. We expect EPF to be a supportive tool for assisting the lattice technique to increase the effort on the cryptanalysis of RSA. As a conclusion, we would like to point out that with the continuous improvements in computational capability, the security levels are expected to be higher with the assistance of EPF, and the security analysis should be considered more carefully.

## Figures and Tables

**Table 1:** The improvement of EPF on *p* + *q*, where *p* and *q* are balanced.

*n*	512	1024	2048
log_2_⁡(*D*_*p*_ − *D*_*q*_)	248.476	504.626	1016.551

*t* (in average)	146.229	295.772	594.103
log_2_⁡(*h*_*t*_)	247.161	503.04	1015.201
log_2_⁡(*h*_*t*+1_)	250.12	506.21	1018.14

**Table 2:** The improvement of EPF on *p* + *q*, where *p* and *q* are balanced.

Balanced Modulus *N* = *pq*	*n* = 512	*n* = 1024	*n* = 2048
$\log_{2}\left(\left(p+q\right)-2\sqrt{N}\right)$	248.476	504.626	1016.551
log_2_⁡((*p* + *q*) − (*p*_*E*_ + *q*_*E*_))	247.185	503.294	1015.248

**Table 3:** The improvement between each attack.

Attacks	Boundary	Complexity
Wiener Attack	*d* < *N* ^1/4^	Polynomial time
V-T Extension	*d* < *N* ^1/4^2^*r*^	Exhaustive search for *U* and *V* of 2*r* + 8 bits (see (13))
Proposed Improvement (Step 1)	*d* < *N* ^1/4^2^*r*^	Exhaustive search for 2*r* + 2 bits (see (24))
Applying EPF (Step 2)	*d* < *N* ^1/4^2^*r*^	Exhaustive search for 2*r* − 6 bits (see (48))
